# Application of real-time detection transformer based on convolutional block attention module and grouped convolution in maize seedling

**DOI:** 10.3389/fpls.2025.1672746

**Published:** 2025-10-10

**Authors:** Yunlong Wu, Shouqi Yuan, Yue Tang, Lingdi Tang

**Affiliations:** ^1^ Research Center of Fluid Machinery Engineering and Technology, Jiangsu University, Zhenjiang, China; ^2^ Data and Informatization Department, Jiangsu University, Zhenjiang, China; ^3^ Faculty of Agricultural Engineering of Jiangsu University, Zhenjiang, China

**Keywords:** maize seedling, UAV remote sensing, CBAM, grouped convolution, real-time detection

## Abstract

**Introduction:**

The intelligent detection and counting of maize seedlings constitute crucial components in future smart maize cultivation and breeding. However, the detection of maize seedlings in field environments faces substantial challenges due to their relatively small target size and the complex environment of the farmland.

**Methods:**

This study proposed an improved detection model named CBAM-RTDETR. Based on the original feature extraction backbone network of RT-DETR, the model introduced the CBAM module and grouped convolution.

**Results:**

The CBAM-RTDETR model achieved a mean Average Precision at 0.5 IoU threshold (mAP0.5) of 92.9%, a mean Average Recall (AR) of 64.4%, and a Frames Per Second (FPS) of 87f/s on the test dataset, all of which are better than the comparison model.

**Discussion:**

The proposed model strengthened the shallow edge detail information of the seedlings and increased the feature diversity, effectively addressed the challenges of real-time and accurate identification of maize seedlings in UAV remote sensing images.

## 1 Introduction

As one of China’s primary grain crops, maize occupies a substantial cultivation area, accounting for 37.1% of the total grain cultivation area nationwide, predominantly in Northeast, North, Northwest, and Southwest China, and making substantial contributions to national grain yield increases. Therefore, maintaining high and stable maize yields remains imperative for ensuring national food security. The emergence rate during the maize seedling stage is a critical parameter influencing planting, cultivation, and subsequent yield, and serves as a key evaluation metric for assessing the quality of maize varieties (National Bureau of Statistics of China(NBSC), 2024). The traditional manual monitoring and counting methods for maize seedlings are time-consuming, cumbersome and error-prone, particularly in open-field environments where human intervention becomes impractical. Therefore, achieving accurate identification of maize seedlings under field conditions remains of paramount importance.

In recent years, UAVs have been rapidly developed and widely used in the field of agricultural observation (Aboelyousr et al., 2025). UAV remote sensing, characterized by low cost, simple operation and robust anti-interference capabilities, can be equipped with various sensors including visible-light RGB, multispectral, hyperspectral, and thermal infrared cameras. This technology enables efficient and non-destructive acquisition of crop growth status information in field conditions. Particularly, utilizing UAV remote sensing for monitoring growth data during the seedling stage represents an optimal solution for precision agriculture (Zeng et al., 2025). UAV remote sensing often contains many small target objects in the captured remote sensing images due to the shooting height and angle, especially in complex agricultural scenes. These factors impose significant constraints on the effective analysis and identification of UAV-captured images in farming environments. Consequently, achieving accurate and efficient processing and identification of small-sized crop objects in UAV remote sensing data remains both crucial and challenging for precision agricultural applications.

Currently, researchers worldwide have achieved notable progress in employing deep learning methods to process crop image data and conduct crop identification and counting studies. Chen et al. replaced the C2f block of the backbone network in YOLOv8n with the Swin-conv block and combined the ParNet attention module in the backbone and neck parts, thereby proposing an efficient, fast and real-time cabbage seedling counting method (Chen et al., 2024). Tang et al. introduced the Global Attention Mechanism (GAM) based on YOLOv5, which improved feature extraction performance. The mean average precision (mPA) values reached 94.5% and 88.2% for maize seedling detection at unmanned aerial vehicle (UAV) flight altitudes of 15 m and 40 m, respectively, with an average detection speed of 0.025 s per image, realizing the accurate and fast identification of maize seedlings for UAV RGB images under the weed interference condition (Tang et al., 2025). The above research results show that the use of deep learning methods has become an effective and promising approach for crop identification and counting. However, most studies usually use YOLO detector as the basic model of crop seedling object identification. Although these detection models have high detection accuracy, they usually need to manually set the prior box before training, and need to carefully select the appropriate Non-Maximum Suppression (NMS) for post-processing operations, resulting in high computational costs and affecting identification performance (Cao et al., 2023; Li X. et al., 2025; Kumar et al., 2025). In 2020, Facebook proposed DETR, an end-to-end object detection algorithm based on Transformer. DETR reconfigures object detection as a sequence prediction problem, eliminating the need for threshold filtering and non-maximal suppression in the post-processing step of traditional dense detection. However, the large number of parameters in DETR leads to its high computational cost. Zhao et al. proposed RT-DETR, a real-time end-to-end object detector (Zhao et al., 2024). Although RT-DETR achieves efficient object detection with reduced computational costs, its performance in the field of small object detection still requires improvement. Kong et al. introduced the Effective Small Object Detection Network (EDF-FAM) into the neck network of RT-DETR to enhance the model’s ability to fuse features of small objects, thereby overcoming the difficulty in detecting small objects in high-precision remote sensing images (Kong et al., 2024). Gu et al. improved the speed and efficiency of the model for tomato identification by combining the dilated convolution, the focusing feature downsampler, and the adaptive feature upsampler embedded at the same time into the encoder structure of RT-DETR network (Gu et al., 2024). Wang et al. introduced PConv_Block module based on RT-DETR and proposed PDSI-RTDETR. The model reduced the computational load and improved the fine-grained detection of tomato ripeness, and finally enhanced the detection ability of small objects (Wang S. et al., 2024).

The above research results showed that RT-DETR had some optimization potential in the small object detection. In addition, considering the solution of high view angle and complex background images under UAV remote sensing, the seedling maize object is small and insignificant, which is difficult to detect. Therefore the detection model of RT-DETR based on CBAM and grouped convolution was improved and designed: CBAM-RTDETR. The improved CBAM-RTDETR increased the attention mechanism in both channel and spatial dimensions to the backbone network PResNet50 of feature extraction. By dynamically adjusting the weight of the feature layer in the channel and space dimensions, the key features were enhanced and the influence of noise was reduced, thereby improving the robustness and performance of the model (Woo et al., 2018). On this basis, grouped convolution was introduced to reduce the amount of calculation while ensuring parameter sharing and improving feature diversity, so that the network had stronger feature expression ability (Guo et al., 2024). It was expected that CBAM-RTDETR can improve the detection accuracy of small objects while ensuring the detection and identification rate. Finally, it was verified by experiments that CBAM-RTDETR improved the detection accuracy while ensuring the real-time detection of maize seedlings, which provided technical support for maize cultivation and yield increase.

## 2 Materials and methods

### 2.1 Experimental environment

**Figure 1:**
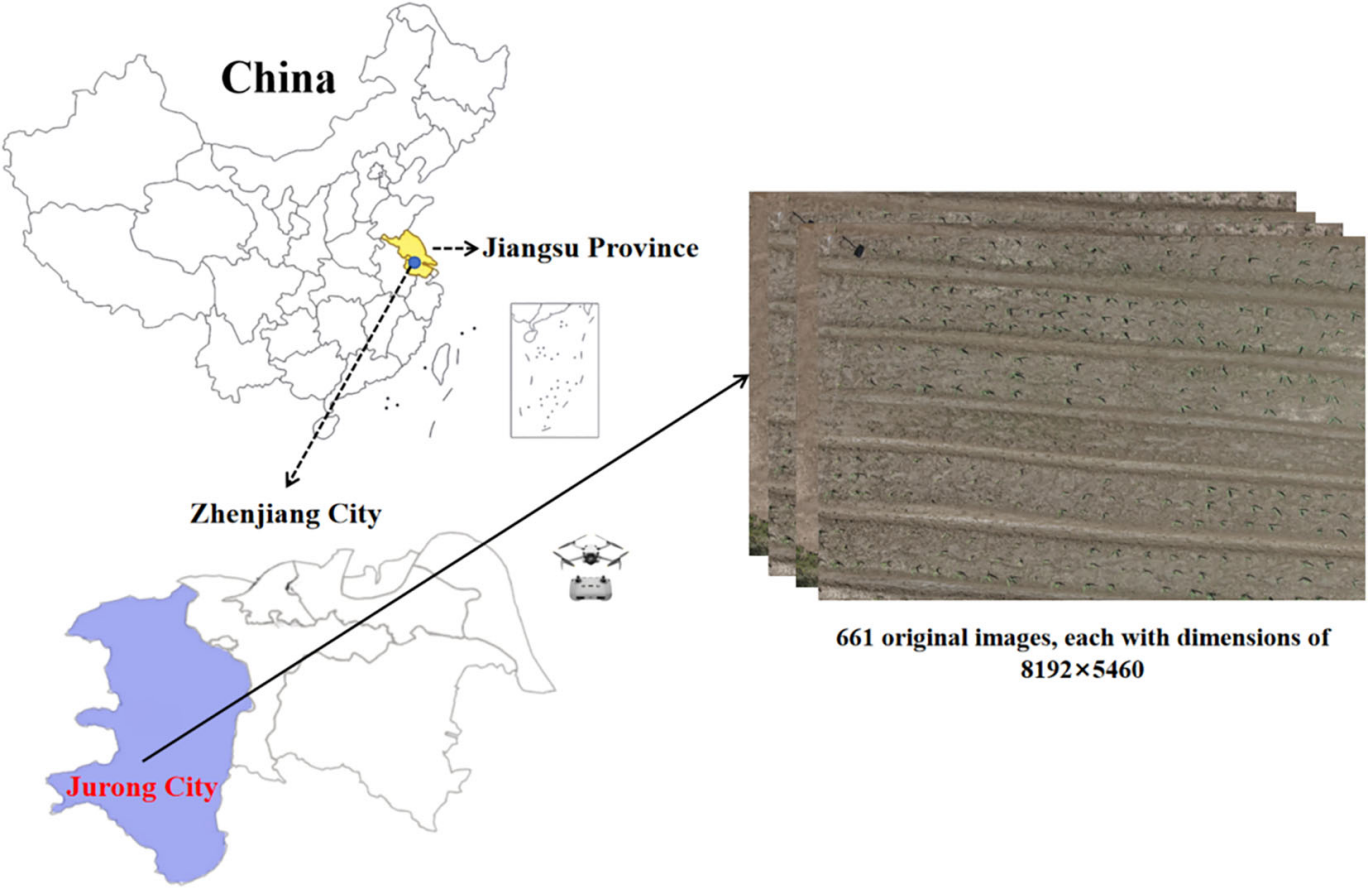
The experimental area map and UAV sampling.

The experimental field was located in Jurong City, Jiangsu Province, China, as shown in **Figure 1**. The soil type was sandy loam. The experimental area was a subtropical monsoon climate. Maize was planted on June 17, 2024, and the variety was Suyu 161. The size of the experimental area was 44 m×56 m experimental field, planting density was 57,000 plants per hectare (ha). The row spacing and plant spacing were 30 cm.

### 2.2 Experimental data acquisition and pre-processing

The UAV image acquisition was conducted on July 4, 2024, 17 days after maize planting, with maize seedlings at the V3-V5 true leaf stage, with a few weeds in the field, and no visible seedling adhesion. The UAV model used for image acquisition was DJI Mini 4 Pro (SZ DJI Technology Co, Ltd. Beijing, China) with a 1.3-inch 48-megapixel visible light sensor, as shown in **Figure 1**. The acquisition took place at 11:30 PM under sunny and windless weather. The route altitude was 10m and the overlap rate was 50%, and a total of 661 original images of maize seedlings with a pixel resolution of 8192×5460 were acquired. Then, using the Pillow 9.5.0 image processing library, the raw images were cut to 640×640 pixels.

**Figure 2:**
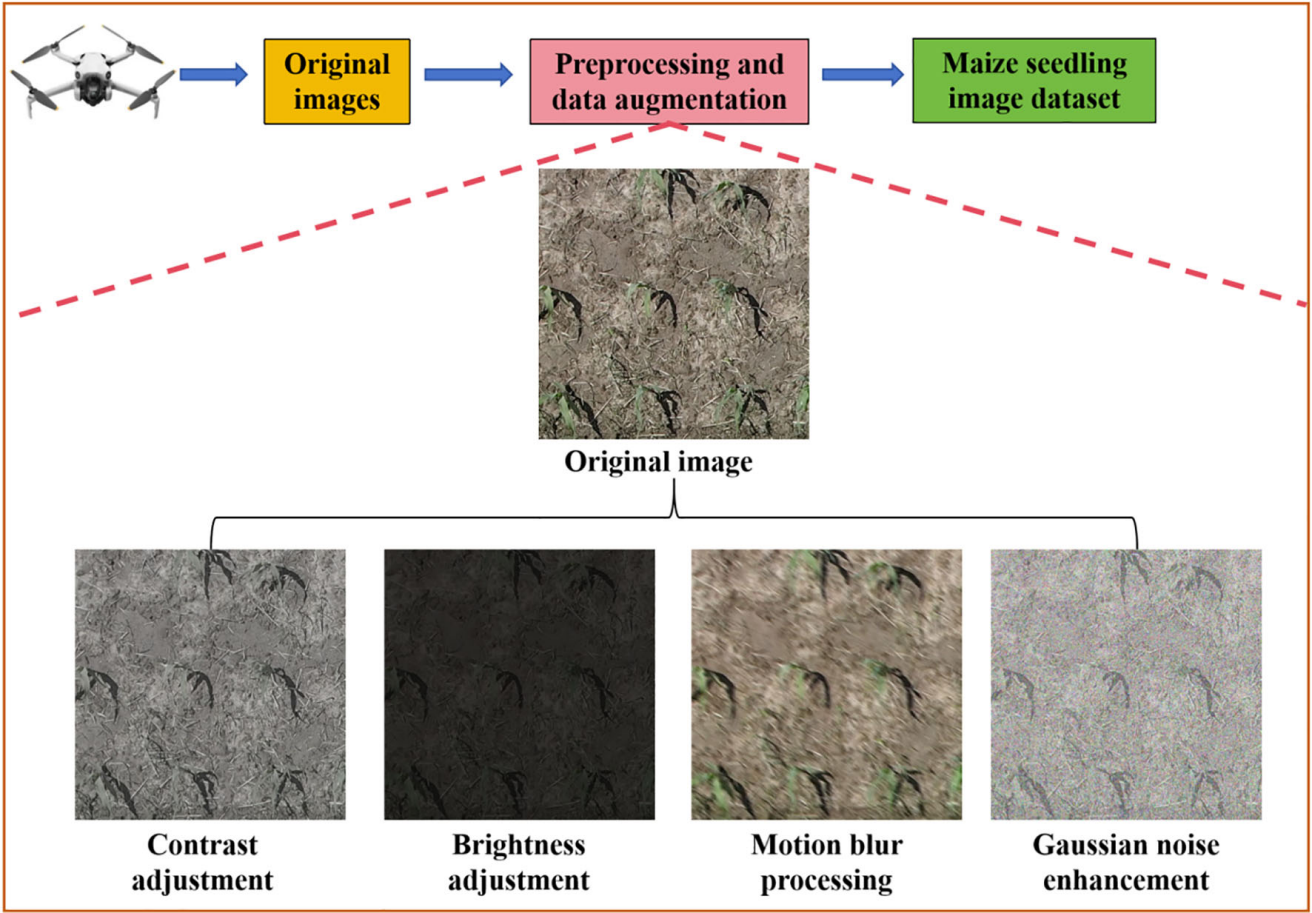
The method for obtaining the dataset of maize seedlings.

The Labelme software (https://github.com/labelmeai/labelme) was used to mark the maize seedlings in the images and the datasets were divided into training, evaluation and test datasets in the ratio of 7:2:1. Among them, the training and evaluation datasets were used for model training, and the test datasets was used for model effect testing. In addition, in order to improve the generalization ability of the model and make up for the limited number of datasets. Using the image enhancement method in the Pillow 9.5.0 image processing library, the contrast, brightness and color of the training and evaluation datasets were adjusted, and motion blur and Gaussian noise were added. Finally, 3360 training datasets, 960 evaluation datasets and 480 test datasets were obtained. **Figure 2** illustrated the acquisition method of the maize seedling dataset in this study, and the image examples after being processed through different data augmentation methods.

From the original remote sensing images of 480 test datasets, 20 original images with a resolution of 8192lutiono pixels were selected (the 20 images were randomized and did not have any obvious overlapping areas). Then these 20 images were cropped to 5440pedping pixels, which was used as the dataset for the counting test. Through manual counting, a total of 5005 maize seedlings were obtained from the counting test dataset.

### 2.3 RT-DETR object detection architecture

RT-DETR model is an end-to-end real-time object detection model based on Transformer. It mainly consists of a backbone, an efficient hybrid encoder, and a TransFormer decoder with an auxiliary prediction header (Zhao et al., 2024). The features of the last three stages output from the backbone network are used as inputs to the encoder. The efficient hybrid encoder transforms the multi-scale feature through Attention-based Intra-scale Feature Interaction (AIFI). Simultaneously, the CNN-based Cross-scale Feature Fusion (CCFF) module transforms these features into an image feature sequence. Subsequently, the minimum uncertainty query module selects a certain number of encoder features to serve as initial object queries for decoder input. The auxiliary prediction head module of the decoder continuously iterates and optimizes object queries, and finally generates categories and object boxes.

The RT-DETR model has an efficient hybrid encoder and eliminates NMS post-processing, which has great potential in the field of real-time object detection (Wang S. et al., 2024; Wu et al., 2025; Zhang C. et al., 2025). Therefore, this study focuses on design and improvement of the RT-DETR model.

### 2.4 Improved CBAM-RTDETR object detection network

The identification of maize at the seedling stage is a small object detection task, especially the maize seedlings under the UAV remote sensing image as a smaller object, which increases the difficulty of detection and identification (Jia et al., 2024; Wei et al., 2024; Liu et al., 2024). In order to improve the feature extraction ability of RT-DETR model for maize seedlings, a new network model CBAM-RTDETR is proposed by introducing the BottleNeck module in the feature extraction backbone network PResNet of RT-DETR to CBAM attention mechanism and grouped convolution. Specifically, the CBAM mechanism enhances calibration precision through channel-wise and spatial weight modulation across feature layers, while Grouped Convolution enriches feature representation diversity through parameter-efficient grouped computation paradigms.

**Figure 3:**
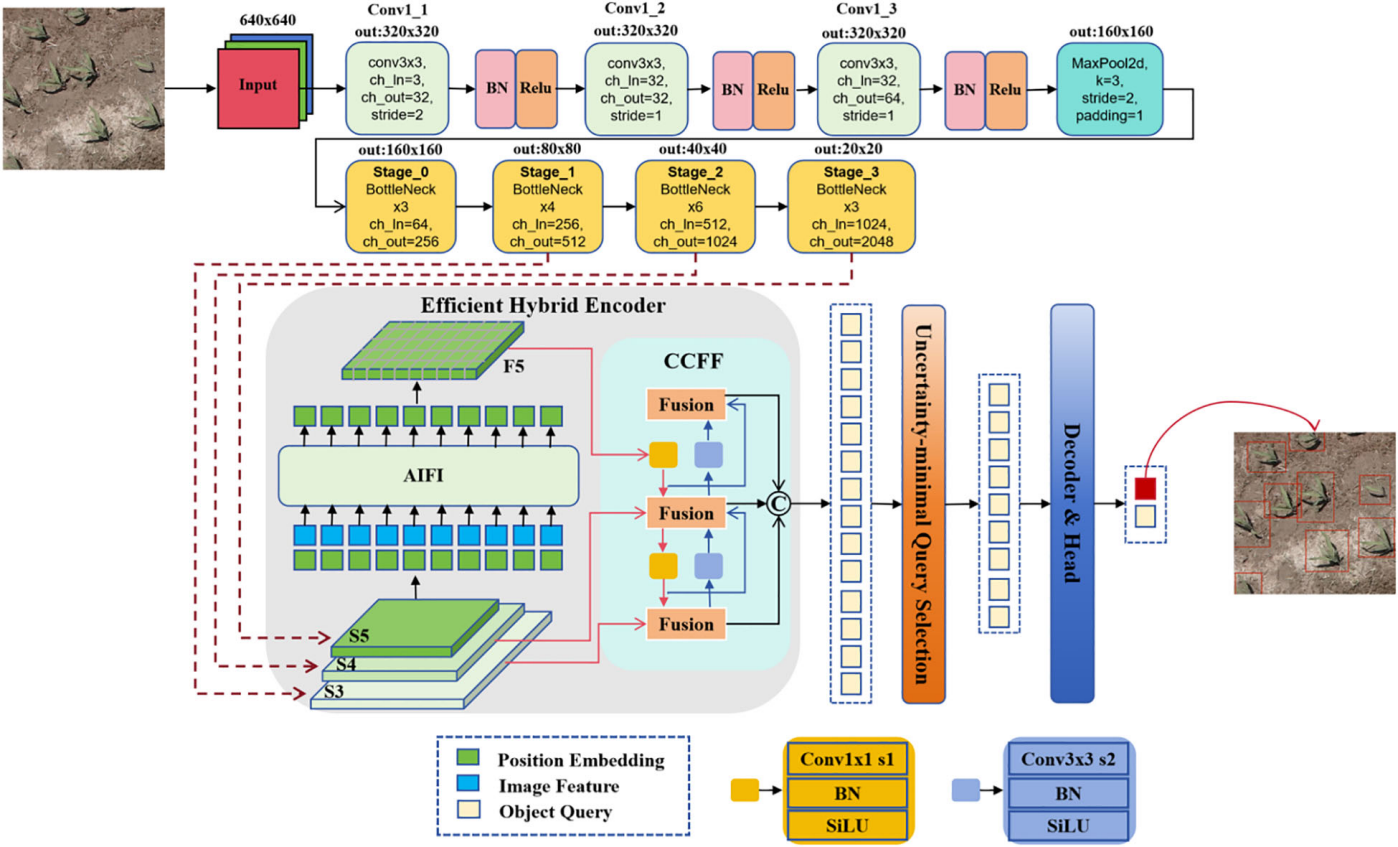
CBAM-RTDETR model network structure. The feature extraction backbone first passed through three standard convolutions, batch normalization processing, and ReLU activation function. Then, it underwent a max pooling operation to reduce the size of the feature layer, and finally passed through 4 stages. The outputs of the last three stages were passed to the encoder. The CBOM-RTDETR model introduced the CBAM attention mechanism and grouped convolution in each Stage to enhance key features and reduce the influence of noise, while also reducing the computational load.

The backbone input of the improved CBAM-RTDETR model is a remote sensing maize seedling image with H×W×3 . It first passes through three convolutional layers, which control the feature extraction receptive field and output feature sizes through the Kernel Size (K), Stride Size (S), and Patch Size (P). The first convolutional layer reduces the image size to $\frac{H}{2}\times \frac{W}{2}$ . Each convolutional layer is followed by a Batch Normalization and ReLU activation function, followed by a max-pooling layer that further reduces the image size to $\frac{H}{4}\times \frac{W}{4}$ . Then four stages of BottleNeck modules process these features, with each module executing n iterative transformations, and for the last three BottleNeck modules, the image undergoes a downsampling, reducing the size to half of its original size. Finally, the output of the last three BottleNeck modules is used as the input of the Efficient Hybrid Encoder (EHC), and the network structure is shown in **Figure 3**.

Each BottleNeck module structure consists of three parts: the primary path, the shortcut, and the attention mechanism with feature fusion operations. The primary path mainly contains three convolutional layers, The first layer is a 1x1standard convolution operation to achieve dimensionality reduction, the second layer is a grouped convolution, assuming that the standard convolution of the convolution kernel is $h\times w\times c_{1}$ , the number of convolution kernels is $c_{2}$ , the parameter quantity is shown in Equation 1. Using grouped convolution, the number of groups is *g*, and the number of parameters is reduced to $\frac{1}{g}$ of the original, as shown in Equation 2. Grouped convolution not only reduces the number of parameters, but also increases the feature layer network width and expression ability through multi-group learning. The third layer is also a 1x1 standard convolution operation, which realizes the dimension elevation operation of the feature layer.


(1) $$Params=h\times w\times c_{1}\times c_{2}$$

(2) $$Params=h\times w\times \frac{c_{1}}{g}\times \frac{c_{2}}{g}\times g=h\times w\times c_{1}\times c_{2}\times \frac{1}{g}$$

The shortcut mainly is mainly implemented to adjust dimensions and channels. When the first operation of each BottleNeck module, the input and output channels are inconsistent with the dimensions, using the average pooling and 1×1 convolution operation to adjust the feature dimension and the number of channels, rather than the first operation when the dimension is consistent, the 1×1 convolution operation is used for identity mapping.

**Figure 4:**
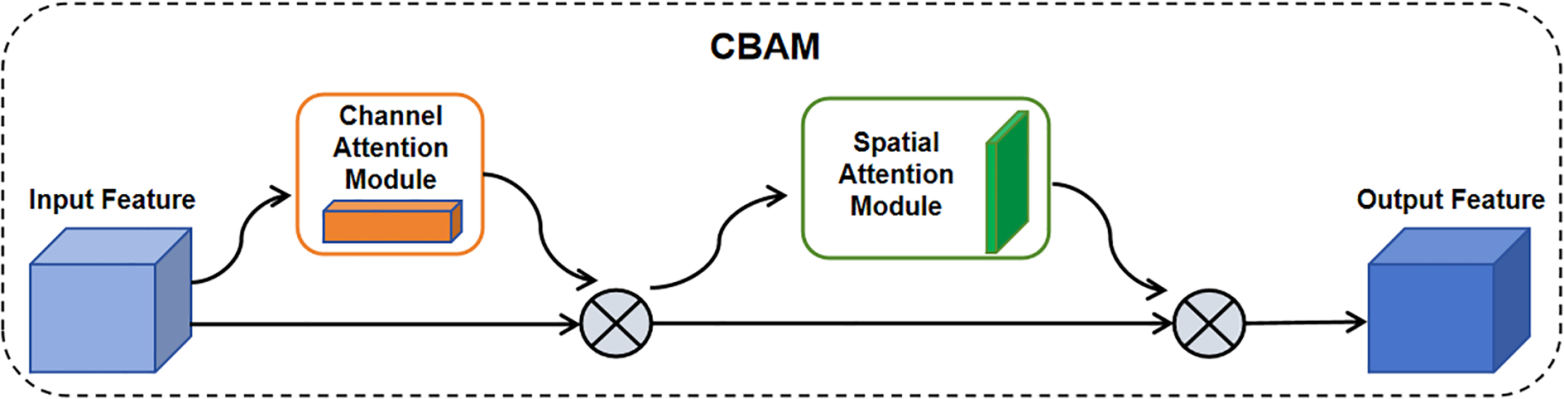
CBAM network structure. It consisted of channel attention module and spatial attention module. The input features passed through the attention module and then were multiplied element-wise with the input features. The CBAM-RTDETR model added the CBAM module to the feature extraction backbone, establishing the dependency of features in both channels and spaces.

The feature layer of the primary path output goes through a batch normalization process and then enters the CBAM attention mechanism module. The CBAM consists of a channel attention module and a spatial attention module, respectively, which allows the feature layer to attend to important features and suppress unimportant features at both the channel and spatial levels as shown in **Figure 4**. The output of the CBAM is summed up with shortcut and then outputted after passing through the ReLU nonlinear activation function to the next operation or the next Stage.

### 2.5 Training and implementation of CBAM-RTDETR

The server environment for model training is Ubuntu 20.04.6 LTS, NVIDIA GeForce RTX 4090 GPU, 24G video memory, 20-core CPU, 80G memory. The deep learning framework uses PyTorch 2.0.

**Figure 5:**
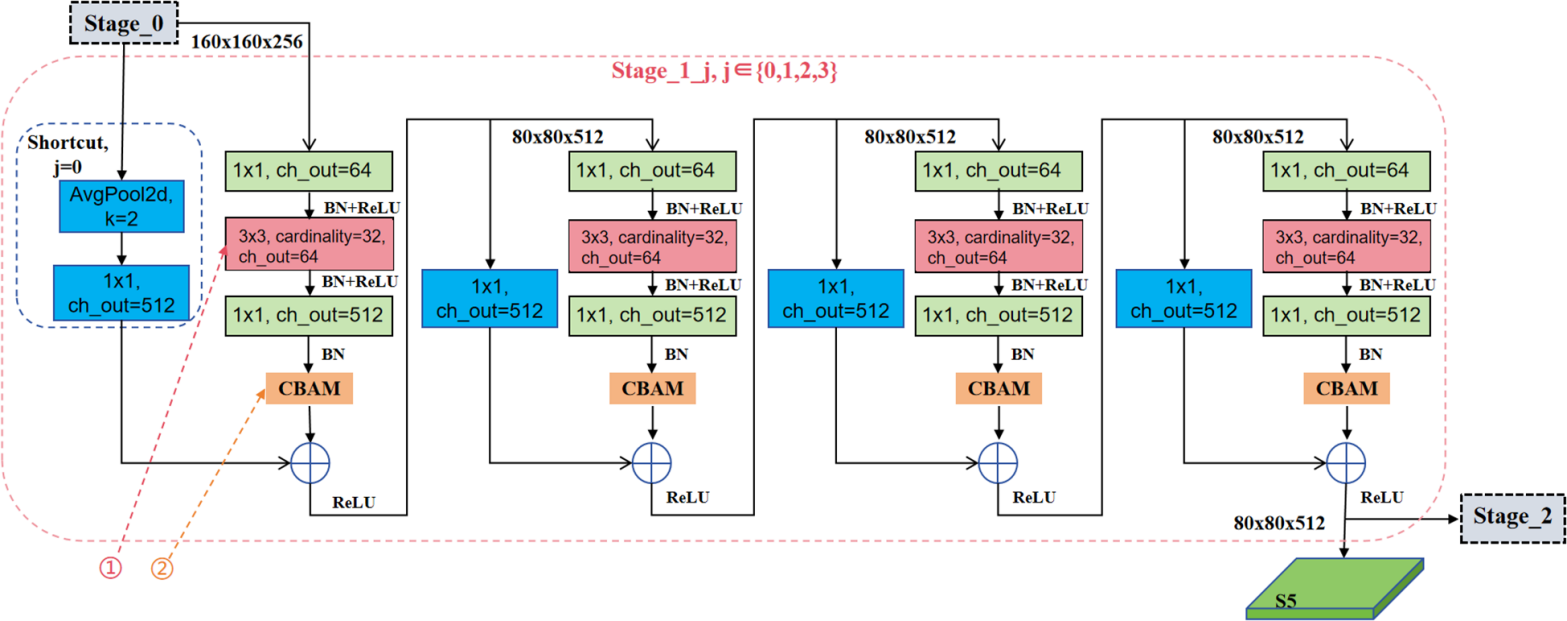
Network structure of Stage_1.

The feature extraction of CBAM-RTDETR backbone input is a 640×640×3 dimensional image. First, it passes through three standard convolutional layers, the first convolutional layer (K = 3, S = 2), realizes the downsampling of the image to 320×320 pixels, and the number of output channels is 32. The latter two convolutional layers have step sizes of S = 1, and the output channels are 32 and 64, respectively. After a layer of max-pooling (K = 3, S = 2, P = 1) operation, the feature layer of 160×160×64 dimensions were output and used as the input of Stage_0. Stage_1, Stage_2, and Stage_3 all go through a downsampling in the first BottleNeck module, and the feature map size is reduced to half of its original size. The number of BottleNeck operations in Stage_i,i∈{0,1,2,3} is n∈{3,4,6,3} , and the number of feature channels output is ch_out∈{256,512,1024,2048} . Taking Stage_1 as an example, its network structure is shown in **Figure 5**, where ① is the grouped convolution and ② is the CBAM module. The output of Stage_0 is used as the input of Stage_1, which contains four BottleNeck operations, denoted as Stage_1_j,j∈{0,1,2,3} , where Stage_1_0 undergoes a shortcut operation to realize downsampling. The output of Stage_1 is used as the input of the high-efficiency hybrid encoder S5 as well as Stage_2. The other hyperparameter settings for the feature extraction backbone, encoder and decoder in the CBAM-RTDETR model implementation are shown in **Table 1**.

**Table 1:** Details of CBAM-RTDETR and the compared methods.

Methods	Backbone	Optimizer	Hyperparameters for training
YOLOv5	CSPDarknet53	SGD	Backbone: CSPDarknet; Learning rate: initial was 0.001, min was 0.00001, decay type was “Cosine Decay”; Optimizer: SGD (momentum=0.937,weight decay=0.0005); Pre-trained: False, Seed = 11.
YOLOv7	CSPDarknet53	SGD	Learning rate: initial was 0.001, min was 0.00001, decay type was “Cosine Decay”; Optimizer: SGD (momentum=0.937,weight decay=0.0005); Pre-trained: False, Seed = 11.
YOLOv8	CSPDarknet53	SGD	Learning rate: initial was 0.001, min was 0.00001, decay type was “Cosine Decay”; Optimizer: SGD (momentum=0.937,weight decay=0.0005); Pre-trained: False, Seed = 11.
RT-DETR	PResNet50	AdamW	Encoder: HybridEncoder (in_channels:[512, 1024, 2048], feat_strides: [8, 16, 32], hidden_dim=256, nhead=8, activation function=‘gelu’); Decoder: RTDETRTransformer (feat_channels: [256, 256, 256], feat_strides: [8, 16, 32], hidden_dim=256, num_levels=3, num_queries=300, num_decoder_layers: 6, num_denoising: 100).
CBAM-RTDETR	PResNet50+GC+CBAM	AdamW	Backbone: PResNet50+CBAM+GC (depth=50, out_channels= [256, 512, 1024, 2048], reduction_ratio=16, cardinality=32); Encoder: HybridEncoder (in_channels:[512, 1024, 2048], feat_strides:[8, 16, 32], hidden_dim=256, nhead=8, activation function=‘gelu’); Decoder: RTDETRTransformer (feat_channels: [256, 256, 256], feat_strides: [8, 16, 32], hidden_dim=256, num_levels=3, num_queries=300, num_decoder_layers: 6, num_denoising: 100).

The model was trained without pre-training weights, and a total of 130 epochs were trained, with a batch size of 8. The AdamW optimizer was used in the training to improve the convergence efficiency. The initial learning rate was set to 0.001 and the minimum learning rate was set to 0.00001.

### 2.6 Model performance evaluation

#### 2.6.1 The compared methods

In order to demonstrate the efficiency of the CBAM-RTDETR model, comparative tests were conducted to compare it with the following object detection methods, respectively. The training and evaluation datasets for all networks are 3360 and 960 RGB images of 640×640×3 dimensions obtained in Section 2.2, respectively. The confidence level of all models was set to 0.5, indicating that those with an identification probability of more than 0.5 were considered to be maize seedlings.

(1) Studies have shown that YOLOv5, YOLOv7 and YOLOv8 networks can accurately and efficiently identify and count small object crops under UAV remote sensing images (Akdoğan et al., 2025; Wu et al., 2023; Wei and Wang, 2025; Paul et al., 2024; Du et al., 2025). Therefore, these networks are used in this paper as comparison methods. These networks are trained without pre-training weights, with YOLOv5 using CSPDarknet (Wang et al., 2020) for backbone, YOLOv7 using the tiny version, and YOLOv8 using the YOLOv8-s version. These networks all have three anchors, which are [10,13,16,30,33,23], [30,61,62,45,59,119] and [116,90,156,198,373,326]. Other training hyperparameters are shown in **Table 1**.

(2) CBAM-RTDETR optimizes the feature extraction network based on RT-DETR, so it is necessary to use RT-DETR as a comparison network. The feature extraction network of RT-DETR in the experiments of this paper adopts the standard PResNet (He et al., 2016), where depth is set to 50 and stage is set to 4, and the output of the last three stages is used as the input of efficient hybrid encoder.

#### 2.6.2 Evaluation metrics

In order to validate the performance of the CBAM-RTDETR model, the metrics based on Precision, Average Recall (AR), mean Average Precision (mAP) and FPS (Frames Per Second) are mainly used for quantitative evaluation of all network models. The dataset applied to all models were: the 3360 training datasets, 960 evaluation datasets and 480 test datasets obtained in Section 2.2. The equations of Precision, Recall and mPA are as follows:


(3) $$\text{Precision }=\;\frac{\text{TP}}{\text{TP}+\text{FP}}$$

(4) $$\text{Recall}=\frac{\text{TP}}{\text{TP}+\text{FN}}$$

(5) $$\text{Average Recall (AR) }=\;\frac{1}{\text{N}}\sum_{i=1}^{N}{\text{Recall}}_{i}$$

(6) $$\text{mAP}=\frac{\sum_{1}^{\text{n}}\int_{0}^{1}\text{Precision(Recall)d(Recall)}}{n}$$

In Equations 3 and 4, TP, FP and FN represent the number of true positives, false positives and false negatives, respectively. In Equation 5, N represents the number of all samples in the network model. In Equation 6, AP represents the area under the precision recall curve (P-R curve), and mAP represents the mean value of different categories of AP. In this experiment, there was only one category of maize seedlings, so n=1, where mAP_0.5_ represents the mean value of mAP when the IOU threshold is 0.5, mAP0.75 represents the mean value of mAP when the IOU threshold is 0.75, the mAP_0.50-0.95_ represents the mean value of mAP at different IOU thresholds (IOU: 0.5-0.95, step size 0.05). In addition, FPS represents the number of frames per second that the object identification network can process images, which is a performance metric to evaluate the speed of the object detection algorithm.

## 3 Results and analysis

### 3.1 Model training results

**Figure 6:**
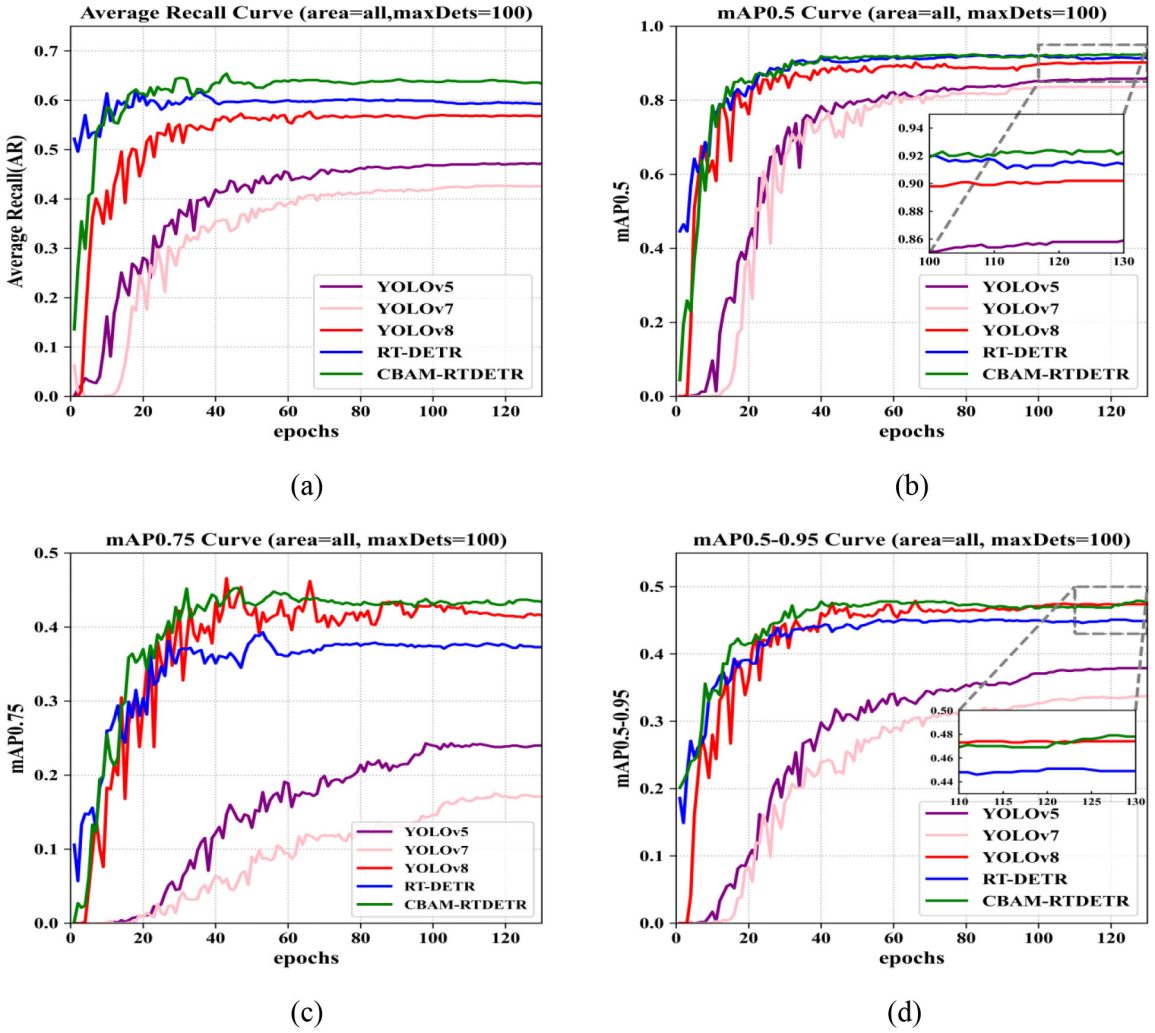
Comparison of evaluation metrics for different detection models: **(A)** average recall (AR) curves; **(B)** mAP_0.5_ curves; **(C)** mAP_0.75_ curves; **(D)** mAP_0.5-0.95_ curves.

In order to validate the effect of the improved CBAM-RTDETR, a total of six object identification models are constructed in this paper, namely, YOLOv5, YOLOv7, YOLOv8, RT-DETR, and CBAM-RTDETR. **Figure 6** demonstrates the AR, mAP_0.5_, mAP_0.75_, and mAP_0.50-0.95_ curves of all models on the evaluation dataset. During the training process, the number of experimental training sessions for all network models was set to 130, and the evaluation metrics of each model on the evaluation dataset tended to stabilize, indicating that all network models finally converged on the evaluation dataset. As can be seen in the figure, the CBAM-RTDETR model performs best in AR, mAP_0.5_, mAP_0.75_ and mAP_0.50-0.95_, especially in AR and mAP_0.75_, which are significantly better than other models. In the AR and mAP_0.5_ metrics, CBAM-RTDETR around the first 20 epochs is not dominant, slightly worse than RT-DETR. It may be because the feature extraction backbone of CBAM-RTDETR adds CBAM and grouped convolutional module, pays more attention to the objecting and expression ability of feature extraction, which requires more data, time and computational cost. However, after 20 epochs, the evaluation metrics of CBAM-RTDETR start to outperform other models.

### 3.2 Model performance evaluation

In order to verify the generalization ability of the model and test the identification speed of the model, 480 test datasets of section 2.2 are used on the trained model in the same environment as the training of section 2.5, and the results are shown in **Table 2**. In terms of detection accuracy, the CBAM-RTDETR model has the best performance in mAP_0.5-0.95_, mAP_0.5_, mAP_0.75_ and AR metrics, reaching 48.2%, 92.9%, 43.6% and 64.4%, respectively, which is an improvement of 3.1%, 1.6%, 6.3%, and 4.9%, respectively, compared to RT-DETR. The YOLOv7 network model performed the worst on mAP0.5-0.95, mAP0.5, mAP0.75, and AR metrics with 33.8%, 83.7%, 17.1%, and 42.6%, respectively.

**Table 2:** Performance of the model on the test dataset.

Model	mAP(%)			AR(%)	FPS(f/s)
	mAP_0.5-0.95_	mAP_0.5_	mAP_0.75_		
YOLOv5	37.9	85.9	24.0	47.2	65
YOLOv7	33.8	83.7	17.1	42.6	72
YOLOv8	47.4	90.2	41.6	56.9	73
RT-DETR	45.1	91.3	37.3	59.5	**87**
CBAM-RTDETR	**48.2**	**92.9**	**43.6**	**64.4**	**87**

The bold numbers indicate the optimal values of the corresponding indicators.

In terms of real-time detection, both RT-DETR and CBAM-RTDETR achieved FPS of 87 f/s on the test set, which is better than the other models. It indicates that the models can complete detection and identification of maize seedlings on 87 test datasets per second under the experimental conditions. The reason is that although the CBAM attention mechanism is added, which improves the accuracy and increases some computational consumption at the same time, but the computational consumption is controllable. Meanwhile, after replacing the second layer of convolution of the primary path in BottleNeck from ordinary convolution to grouped convolution, it not only improves the feature diversity, but also reduces the parameter quantity and computational cost. The performance of CBAM-RTDETR on FPS is not affected by the offset of the computational complexity of CBAM and grouped convolution.

In order to further validate the identification ability of the proposed CBAM-RTDETR model for maize seedling plants, the counting test dataset in section 2.2 was input into 5 models for identification and counting. The experimental results are shown in **Table 3**. A total of 5005 maize seedlings were identified from 20 remote sensing images, and 4827 were identified by CBAM-RTDETR model, of which 4814 were correctly identified. True positive example (TP): the number of maize correctly identified by the model is 4814, false positive example (FP): the number of maize misidentified by the model is 4827 - 4814 = 13, false counter example (FN): the number of maize that actually exists but has not been identified is 5005 - 4814 = 191. Based on equations (3) and (4), the Precision and Recall of the CBAM-RTDETR model were determined to be 99.73% and 96.18% respectively, which are superior to other comparison models.

**Table 3:** Counting results of maize seedlings by models.

Model	Image	Maize seedling	Identification number	Identify correctly	Precision	Recall
YOLOv5	20	5005	4649	4617	99.31%	92.25%
YOLOv7	20	5005	4619	4580	99.16%	91.51%
YOLOv8	20	5005	4757	4732	99.47%	94.55%
RT-DETR	20	5005	4725	4703	99.53%	93.97%
CBAM-RTDETR	20	5005	**4827**	**4819**	**99.83%**	**96.28%**

The bold numbers indicate the optimal values of the corresponding indicators.

### 3.3 Ablation experiments

To further verify the improvement of the proposed feature extraction scheme on the model’s detection performance, several ablation experiments were conducted on the test set. The experiments aimed to evaluate the impact of the introduction of the CBAM module and the grouped convolution module on the model’s performance in feature extraction. Each experiment set the same hyperparameters and used the same training strategy. The experimental results were shown in **Table 4**. It can be seen that the improved modules significantly enhanced the performance of the base model RT-DETR.

**Table 4:** Results of ablation experiments.

Experimental model	mAP(%)			AR(%)	FPS(f/s)
	mAP_0.5-0.95_	mAP_0.5_	mAP_0.75_		
PResNet50(RT-DETR)	45.1	91.3	37.3	59.5	87
PResNet50+CBAM	46.3	92.5	41.1	60.7	84
PResNet50+GC	45.0	91.3	39.0	59.2	**89**
PResNet50+CBAM+GC (CBAM-RTDETR)	**48.2**	**92.9**	**43.6**	**64.4**	87

The bold numbers indicate the optimal values of the corresponding indicators.

The addition of CBAM module focused the key feature information by dynamically balancing the weights of different channels and spaces. It made the feature extraction network for the case of PResNet50+CBAM improve the mAP0.5-0.95, mAP0.5, mAP0.75, and AR metrics compared to the standard PResNet50 (RT-DETR) by 1.2%, 1.2%, 3.8% and 1.2%, respectively. Although the FPS decreased by 3f/s, it was still within a manageable range. After replacing some of the standard convolutions of BottleNeck in PRESNet50 with grouped convolutions (GCs), compared to the standard PRESNet50 (RT-DETR), the mAP_0.5-0.95_ and AR decreased by 0.1% and 0.3%, respectively, the mAP_0.5_ remained unchanged, and the mAP_0.75_ improved by 1.7%, and the FPS also improved to 89 f/s. Grouped convolution reduced the computational density, improves the inference speed, and improved the diversity and expressiveness of the features. So the feature extraction network with PRESNet50+GC has less impact on the model accuracy and improves the real-time performance and FPS of the model.

**Figure 7:**
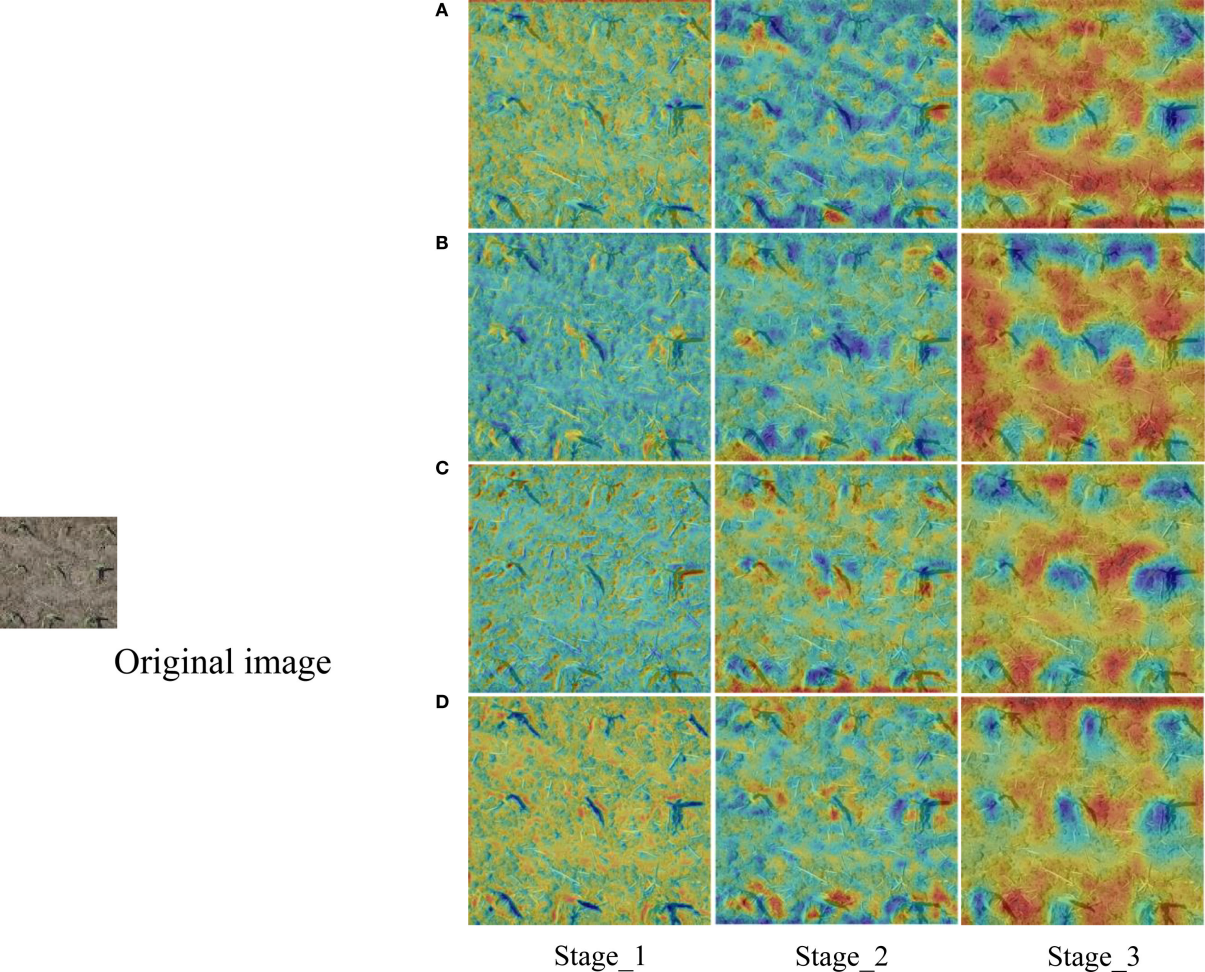
Comparison of thermal maps of the output feature layers from Stage_1 to Stage_3 of each model. **(A)** PResNet50 (RT-DETR); **(B)** PResNet50+CBAM; **(C)** PResNet50+GC; **(D)** PResNet50+CBAM+GC (CBAM-RTDETR).

**Figure 7** presented the feature thermograms of each experimental model after feature extraction backbone. The contrast effect of Stage_3 was particularly obvious. In the thermogram of PResNet50 (RT-DETR), the boundary of dividing each seedling was still unclear. With the increase of CBAM and grouped convolution, the edge thermal distribution of each seedling was more obvious.

After combining the properties of CBAM module and grouped convolution, PResNet50+CBAM+GC as a feature extraction network demonstrates superior performance in both model accuracy and detection speed. PResNet50+CBAM+GC (CBAM-RTDETR) achieved the best results in mAP_0.5-0.95_, mAP_0.5_, mAP_0.75_ and AR, which was significantly improved compared with PResNet50 (RT-DETR), and the FPS was not affected.

## 4 Discussion

### 4.1 Innovative and potential applications

In recent years, YOLO-based object detectors have been frequently used in the identification and detection of UAV remote sensing images, but these detectors eventually generate many overlapping detection frames that require NMS post-processing and slow down the speed of these detectors. In order to remove manual anchors and NMS post-processing operations, an end-to-end detector based on Transformer is proposed. The target detection framework based on transformer usually requires a large amount of computing resources. Additionally, the background of UAV remote sensing images is complex, mainly consisting of small-sized objects. The distribution and scale structure of the objects will change with the shooting angle of the UAV, and even may cause distortion. Facing these challenges, some researchers had attempted to improve the target detection performance of UAV remote sensing images by optimizing the DETR model, but they had encountered significant challenges in balancing accuracy and real-time performance (Gu et al., 2024). For instance, Kong et al. improved the model’s ability to integrate the features of small objects by proposing the ESDNet network based on RT-DETR, thereby enhancing the accuracy of small object detection. However, as the model’s accuracy increased, the real-time performance of the network declined (Kong et al., 2024). Wang et al. proposed the DST-DETR model, which although improved the detection accuracy of safety helmets under foggy conditions, also led to a decrease in the FPS of the model compared to RT-DETR (Wang S. et al., 2024).

In this study, in the face of the challenge of manually counting maize seedlings in the field environment, the UAV is used to take remote sensing images of maize seedlings. Based on Transformer, the feature extraction backbone of RT-DETR is optimized by combining CBAM and grouped convolution, and the CBAM-RTDETR object identification and detection network model is proposed.The CBAM module dynamically learns the weights of different channels and spaces through global average pooling and maximum pooling, highlighting the key information of the object and suppressing noise. Among them, channel attention strengthens shallow details (edge features of maize seedlings), spatial attention suppresses redundant backgrounds of deep features (maize seedlings and soil), which improves the signal-to-noise ratio of the features of the maize seedlings, and significantly improves the detection accuracy of the model at a small computational cost. The second layer convolution of BottleNeck in the backbone of feature extraction is replaced by grouped convolution, and the input channels are divided into multiple groups to realize independent convolution in each group. The number of parameters and calculation is reduced while more independent feature learning paths are introduced, which improves the model detection rate with a small loss of detection accuracy.

By comparing the experimental results of crop recognition and counting studies in recent years, as shown in **Table 5**, the CBAM-RTDETR model also demonstrates excellent performance in terms of accuracy and speed. For instance, Varela et al. extracted the contour of the corn rows using the Excess Green Index (ExG), and then used the decision tree to perform the classification task between corn and non-corn, thereby achieving the recognition of corn. Although machine learning was used to achieve the recognition and counting of corn, with an accuracy rate of 96%, this method was not an end-to-end architecture. It relied on the combination of multiple models, which had high operational costs and latency (Varela et al., 2018). Wu et al. proposed an effective method for large-scale counting of rice seedlings based on UAV visible light remote sensing and Simplified VGG-16. However, the average accuracy rate was only 93%, and the real-time performance of the model was not considered. This method had significant limitations in terms of application and promotion (Wu et al., 2019). Le et al. developed an online corn counting network for near-ground equipment such as manned carts or tractors based on the YoloV3 network and the Kalman filter. The accuracy of this network exceeded 98%, and the average frame rate reached 47 f/s. However, if it is applied to large-scale farmland scenarios, it will be a huge workload for the near-ground equipment that carries this network (Le W. et al., 2022). Liu et al. used UAVs to obtain RGB images of corn seedlings. Then they used the Harris corner detection model and the Faster R-CNN model to detect and count the seedlings. Among 5824 corn seedlings, the recognition accuracy of the two models were 99.78% and 98.45% respectively (Liu et al., 2022), both lower than the counting accuracy of CBAM-RTDETR at the same scale.

**Table 5:** Recent research results on crop recognition and counting comparison.

Sampling method	Research object	Sampling height	Resolution	Method	Performance	Reference
RGB/UAV	corn plant	10m	6000 × 4000	Decision tree	Overall accuracy=96%	Varela et al. (2018)
RGB/UAV	Rice seedling	20m	5427 × 3648	Simplified VGG-16	Accuracy > 93%, R^2^ = 0.94	Wu et al. (2019)
Camera on a cart	corn	0.5m	3840 × 2160	YoloV3 + Kalman filter	Accuracy > 98%, FPS=47f/s	Le et al. (2021)
RGB/UAV	Maize seedling	30m	6000 × 4000	Harris corner	Accuracy =99.78%	Liu et al. (2022)
RGB/UAV	Maize seedling	30m	6000 × 4000	Faster R-CNN	Accuracy =98.45%	Liu et al. (2022)

Furthermore, through ablation experiments, it could be found that the CBAM-RTDETR model proposed in this study, through the introduction of CBAM and grouped convolution in the feature extraction backbone network, comprehensively balanced the accuracy and rate, significantly improved the detection accuracy while guaranteeing the detection rate and real-time performance of the model. Beyond its primary application in maize seedling detection, the model exhibits significant potential for extension to other agricultural applications including weed identification, crop pest identification, fruit picking, edge computing and robot automatic picking.

### 4.2 Limitations

Although the proposed CBAM-RTDETR model shows good performance in detection accuracy and real-time performance, further improvement and extensive field tests are needed to verify its robustness under different operating conditions. For example, the sample images collected did not cover sufficient environmental noise (such as field weeds or debris, foggy weather, etc.) to verify the anti-interference ability of the model. In addition, the flight altitude analysis was limited to 10-meter captures, without comparative studies at other altitudes (e.g., 15m, 30m). Then, while outperforming RT-DETR in detection accuracy, the model showed no significant improvement in real-time performance. To address these limitations, future work needs to be tested in various environments, including different weather conditions, longer observation periods, more diversified datasets, and images adapted to different resolutions. The latest target detection model based on the YOLO architecture was also continuously enhancing its ability to extract features, and had also improved the detection performance in dense scenarios by optimizing the NMS mechanism (Wang X. et al., 2024; Wang et al., 2025; Li S. et al., 2025; Zhang H. et al., 2025). However, it has still not been able to overcome the impact caused by the computational cost of NMS. In the future, we will continue to pay close attention to the latest models and development trends in the field of object detection, and constantly compare them with our current models to optimize our models.

## 5 Conclusion

This study addresses the challenging task of maize seedling detection and counting in field environments by proposing CBAM-RTDETR, a UAV remote sensing-based object detection algorithm. The algorithm introduces CBAM attention mechanism and grouped convolution in the feature extraction backbone network of RT-DETR model, which realizes accurate and fast detection of maize seedlings. The CBAM module strengthens the shallow edge detail features of the maize seedlings and suppresses the redundant background information of the deeper features by adjusting the channel and spatial attention weights of the feature layer, guiding the network to focus on the key features in a fast way, which significantly improves the detection accuracy of the model within the acceptable cost of computation. Replacing some of the standard convolutions in the feature extraction backbone network with grouped convolutions enhances the ability to learn local features, and reduces the number of parameters and computation of the model, balancing the accuracy and real-time performance of the model. The experimental results demonstrate that the CBAM-RTDETR model achieves superior performance across all evaluation metrics on the test dataset, with mAP_0.5-0.95_, mAP_0.5_, mAP_0.75_, and AR reaching 48.2%, 92.9%, 43.6%, and 64.4% respectively. Compared to the baseline RT-DETR model, these results represent improvements of 3.1%, 1.6%, 6.3%, and 4.9% for each corresponding metric. In addition to the FPS performance, CBAM-RTDETR is the same as the RT-DETR model, which can realize the detection and identification of maize seedlings from 87 remote sensing images in one second, showing its excellent real-time performance.

The proposed model effectively addresses the challenges of real-time maize seedling detection and identification of maize seedlings in UAV remote sensing imagery, particularly overcoming difficulties caused by small object sizes and complex background interference, while maintaining balanced performance in both detection accuracy and real-time performance. Although the model proposed in this paper shows good results, it also shows the application potential in certain scenarios. However, the complex weather environment and remote sensing images with different resolutions in reality will be a greater challenge. There are also studies to optimize the computational complexity of the feature extraction module and improve the real-time performance of the model. All of these will be the future work direction, which will further promote the comprehensiveness and applicability of the model.

## Data Availability

The raw data supporting the conclusions of this article will be made available by the authors, without undue reservation.

## References

[B1] Aboelyousr M. Sayegh E. S. Ahmed S. (2025). Multi-criteria decision support model for selecting the appropriate construction management firm. Int. J. Construction Manage. 25, 552–561. doi: 10.1080/01446190902759009

[B2] Akdoğan C. Özer T. Oğuz Y. (2025). PP-YOLO: Deep learning based detection model to detect apple and cherry trees in orchard based on Histogram and Wavelet preprocessing techniques. Comput. Electron. Agric. 232, 110052. doi: 10.1016/j.compag.2025.110052

[B3] Cao J. Bao W. Shang H. Yuan M. Cheng Q. (2023). GCL-YOLO: A ghostConv-based lightweight YOLO network for UAV small object detection. Remote Sens. 15, 4932. doi: 10.3390/rs15204932

[B4] Chen X. Liu T. Han K. Jin X. Wang J. Kong X.. (2024). TSP-yolo-based deep learning method for monitoring cabbage seedling emergence. Eur. J. Agron. 157, 127191. doi: 10.1016/j.eja.2024.127191

[B5] Du S. Yang Y. Yuan H. Cheng M. (2025). Application of deep learning for real-time detection, localization, and counting of the Malignant invasive weed Solanum rostratum Dunal. Front. Plant Sci. 15. doi: 10.3389/fpls.2024.1486929, PMID: 39944948 PMC11814178

[B6] Gu Z. Ma X. Guan H. Jiang Q. Deng H. Wen B.. (2024). Tomato fruit detection and phenotype calculation method based on the improved RTDETR model. Comput. Electron. Agric. 227, 109524. doi: 10.1016/j.compag.2024.109524

[B7] Guo H. Li Y. Chen L. Wang J. Ma Q. (2024). Source-load extreme scenario identification method based on residual grouped convolutional neural network and multilevel attention mechanism. Power Grid Technol. 49, 459–469. doi: 10.13335/j.1000-3673.pst.2024.0688

[B8] He K. Zhang X. Ren S. Sun J. (2016). “Deep residual learning for image identification,” in Proceedings of the IEEE conference on computer vision and pattern identification. 770–778. Las Vegas, NV, USA: IEEE.

[B9] Jia Y. Fu K. Lan H. Wang X. Su Z. (2024). Maize tassel detection with CA-YOLO for UAV images in complex field environments. Comput. Electron. Agric. 217, 108562. doi: 10.1016/j.compag.2023.108562

[B10] Kong Y. Shang X. Jia S. (2024). Drone-DETR: efficient small object detection for remote sensing image using enhanced RT-DETR model. Sensors 24, 5496–5496. doi: 10.3390/s24175496, PMID: 39275406 PMC11397902

[B11] Kumar N. Sharma A. Kumar A. Singh R. Singh S. K. (2025). Cattle verification with YOLO and cross-attention encoder-based pairwise triplet loss. Comput. Electron. Agric. 234, 110223. doi: 10.1016/j.compag.2025.110223

[B12] Le W. Lirong X. Lie T. Huanyu J. (2021). A convolutional neural network-based method for corn stand counting in the field. Sensors 21, 507–507. doi: 10.3390/s21020507, PMID: 33450839 PMC7828297

[B13] Li S. Chen Z. Xie J. Zhang H. Guo J. (2025). PD-YOLO: a novel weed detection method based on multi-scale feature fusion. Front. Plant Sci. 16. doi: 10.3389/fpls.2025.1506524, PMID: 40265119 PMC12011770

[B14] Li X. Wang F. Guo Y. Liu Y. Lv H. Zeng F.. (2025). Improved YOLO v5s-based detection method for external defects in potato. Front. Plant Sci. 16. doi: 10.3389/fpls.2025.1527508, PMID: 40041023 PMC11876418

[B15] Liu Z. Sun C. Wang X. (2024). DST-DETR: image dehazing RT-DETR for safety helmet detection in foggy weather. Sensors 24, 4628. doi: 10.3390/s24144628, PMID: 39066026 PMC11280984

[B16] Liu S. Yin D. Feng H. Li Z. Xu X. Shi L.. (2022). Estimating maize seedling number with UAV RGB images and advanced image processing methods. Precis. Agric. 23, 1604–1632. doi: 10.1007/S11119-022-09899-Y

[B17] National Bureau of Statistics of China(NBSC) (2024). Statistical bulletin of national economic and social development of the people’s republic of China in 2023. Chin. Stats. 3), 9–26.

[B18] Paul A. Machavaram R. Ambuj Kumar D. Nagar H. (2024). Smart solutions for capsicum Harvesting: Unleashing the power of YOLO for Detection, Segmentation, growth stage Classification, Counting, and real-time mobile identification. Comput. Electron. Agric. 219, 108832. doi: 10.1016/j.compag.2024.108832

[B19] Tang B. Zhou J. Pan Y. Qu X. Cui Y. Liu C.. (2025). Identification of maize seedling under weed disturbance using improved YOLOv5 algorithm. Measurement 242, 115938. doi: 10.1016/j.measurement.2024.115938

[B20] Varela S. Dhodda R. P. Hsu H. W. Prasad P. V. Assefa Y. (2018). Early-season stand count determination in corn via integration of imagery from unmanned aerial systems (UAS) and supervised learning techniques. Remote Sens. 10, 343–343. doi: 10.3390/rs10020343

[B21] Wang S. Jiang H. Yang J. Ma X. Chen J. Li Z.. (2024). Lightweight tomato ripeness detection algorithm based on the improved RT-DETR. Front. Plant Sci. 15. doi: 10.3389/fpls.2024.1415297, PMID: 39036358 PMC11257922

[B22] Wang C. Y. Liao H. Y. M. Wu Y. H. Chen P. Y. Hsieh J. W. Yeh I. H. (2020). “CSPNet: A new backbone that can enhance learning capability of CNN,” in Proceedings of the IEEE/CVF conference on computer vision and pattern identification workshops, 390–391. Seattle, WA, USA. doi: 10.48550/arXiv.1911.11929

[B23] Wang Y. Ouyang C. Peng H. Deng J. Yang L. Chen H.. (2025). YOLO-ALW: an enhanced high-precision model for chili maturity detection. Sensors 25, 1405–1405. doi: 10.3390/s25051405, PMID: 40096232 PMC11902443

[B24] Wang X. Zhang C. Qiang Z. Liu C. Wei X. Cheng F. (2024). A coffee plant counting method based on dual-channel NMS and YOLOv9 leveraging UAV multispectral imaging. Remote Sens. 16, 3810–3810. doi: 10.3390/rs16203810

[B25] Wei C. Wang W. (2025). RFAG-YOLO: A receptive field attention-guided YOLO network for small-object detection in UAV images. Sensors 25, 2193. doi: 10.3390/s25072193, PMID: 40218706 PMC11991089

[B26] Wei X. Yin L. Zhang L. Wu F. (2024). DV-DETR: improved UAV aerial small object detection algorithm based on RT-DETR. Sensors 24, 7376. doi: 10.3390/s24227376, PMID: 39599152 PMC11598011

[B27] Woo S. Park J. Lee J. Y. Kweon I. S. (2018). “CBAM: Convolutional block attention module,” in Proceedings of the European conference on computer vision (ECCV). 3–19. Munich, Germany.

[B28] Wu D. Wu R. Wang H. Cheng Z. To S. (2025). Real-time detection of blade surface defects based on the improved RT-DETR. J. Intelligent Manufacturing 36, 1–13. doi: 10.1007/s10845-024-02550-9

[B29] Wu J. Xu W. He J. Lan M. (2023). YOLO for penguin detection and counting based on remote sensing images. Remote Sens. 15, 2598. doi: 10.3390/rs15102598

[B30] Wu J. Yang G. Yang X. Xu B. Han L. Zhu Y. (2019). Automatic counting of in *situ* rice seedlings from UAV images based on a deep fully convolutional neural network. Remote Sens. 11, 691. doi: 10.3390/rs11060691

[B31] Zeng F. Wang R. Jiang Y. Liu Z. Ding Y. Dong W.. (2025). Growth monitoring of rapeseed seedlings in multiple growth stages based on low-altitude remote sensing and semantic segmentation. Comput. Electron. Agric. 232, 110135. doi: 10.1016/j.compag.2025.110135

[B32] Zhang H. Xiao P. Yao F. Zhang Q. Gong Y. (2025). Fusion of multi-scale attention for aerial images small-target detection model based on PARE-YOLO. Sci. Rep. 15, 4753. doi: 10.1038/s41598-025-88857-w, PMID: 39922922 PMC11807196

[B33] Zhang C. Yue J. Fu J. Wu S. (2025). River floating object detection with transformer model in real time. Sci. Rep. 15, 9026. doi: 10.1038/s41598-025-93659-1, PMID: 40091136 PMC11911429

[B34] Zhao Y. Lv W. Xu S. Wei J. Wang G. Cui C. Du Y.. (2024). “Detrs beat yolos on real-time object detection,” in Proceedings of the IEEE/CVF Conference on Computer Vision and Pattern Recognition, 16965–16974. Seattle, WA, USA. Available online at: https://arxiv.org/abs/2304.08069.

